# Clinical features of acute and chronic abdominal pain in children admitted to gastroenterology unit

**DOI:** 10.1186/s12876-026-05196-4

**Published:** 2026-08-11

**Authors:** Man Yue, Wei Wang, Kaiping Zhang

**Affiliations:** 1 Department of Gastroenterology, Anhui Provincial Children’s Hospital, Children’s Hospital of Fudan University (Affiliated Anhui Branch), No. 39 Wangjiang East Road, Hefei, Anhui Province 230000 People’s Republic of China; 2 Hemodialysis Center, Hefei BOE Hospital, Teaching Hospital of Shanghai University Medical College, Hefei, Anhui Province 230000 People’s Republic of China; 3 Department of Urology, Anhui Provincial Children’s Hospital, Children’s Hospital of Fudan University (Affiliated Anhui Branch), No. 39 Wangjiang East Road, Hefei, Anhui Province 230000 People’s Republic of China

**Keywords:** Abdominal pain, Clinical features, Chronic gastritis, Acute gastroenteritis

## Abstract

**Background:**

The incidence of abdominal pain in children has been gradually rising. The study aimed to investigate the clinical characteristics of hospitalized children with abdominal pain for making the accurate diagnosis and treatment.

**Methods:**

This study collected the clinical data from a pediatric institution between January 2023 and January 2026. The data primarily included gender, age, body mass index (BMI), intensity, duration of pain, site of pain, common associated symptoms, diagnoses and surgical interventions.

**Results:**

A total of 1025 patients presenting with abdominal pain were analyzed. Among them, there were 625 boys (60.98%) and 400 girls (39.02%), with a median age of this cohort was 8.0 years (5.00, 11.00). The primary diagnoses were chronic gastritis (*n* = 458, 44.68%), acute gastroenteritis (*n* = 321, 31.32%), Henoch-Schönlein purpura (HSP) (*n* = 100, 9.76%), and chronic enteritis (*n* = 44, 4.29%). Most patients (76.39%) had acute pain, and the median duration of pain was 2 months (0.1, 10.00). The pain in most patients localized in the periumbilical region (54.63%). In addition, The most common concomitant symptoms were vomiting (53.46%), diarrhea (22.63%), and fever (22.44%). 668 (65.17%) patients received gastrointestinal endoscope examination. Moreover, subgroup analyses were conducted based on disease types, intensity, gender and age. Statistical differences were found in some variables including age, gender, intensity, duration of pain, site of pain and concomitant symptoms (*P* < 0.05).

**Conclusion:**

Chronic gastritis was the most common disease in children, followed by acute gastroenteritis, HSP and chronic enteritis. Most children suffered from acute periumbilical pain, with vomiting as the most frequent concomitant symptom. Furthermore, significant differences were observed in some clinical characteristics of different subgroup analyses.

## Introduction

Abdominal pain is one of the most common symptons in a hospital emergency department, with its incidence rate being about 11% [1]. The problem of abdominal pain represents one of the major challenges in today’s clinical practice. It is often associated with different abdominal or extra-abdominal diseases. This pain can occur at different abdominal locations, with diverse intensities and propagation, and it is often accompanied by other symptoms.

Abdominal pain is a symptom that requires physician to make an accurate diagnosis in the shortest possible time. With the accumulation of clinical experience and technique development, doctors have become skilled at diagnosing and treatment for people with abdominal pain. However, most diagnostic information in children regarding abdominal pain arise from adult experience. Children often differ from adults in clinical and epidemic features [2]. Compared to adult with gastroesophageal reflux disease (GERD), chronic gastritis, gastric and duodenal ulcers, irritable bowel syndrome (IBS) and chronic pancreatitis (CP), the common pediatric diseases including chronic gastritis, gastroenteritis and inflammatory bowel disease (IBD) often cause acute abdominal pain. Moreover, child has a more possibility of fever, nausea or vomiting [3, 4].

These differences prompt us to report our 3 years’ experience in children and review published literature when broadening a differential diagnosis. Therefore, a retrospective study was performed to describe the causes of acute and chronic abdominal pain, particularly the pain in the abdominal wall, which contributed to recognizing them and providing clinical experience for physician in face of similar situations.

## Methods

### Study design

This study was conducted in accordance with the principles of the Declaration of Helsinki and was reviewed and approved by the Medical Ethics Committee of Anhui Provincial Children’s Hospital (No. EYLL-2025-020). Written informed consents were routinely obtained from their parents or guardians.

A retrospective analysis of patients presenting with abdominal pain was explored in our department during three years, from January 2023 to January 2026. Only patients aged 0 to 18 years presenting with abdominal pain as their chief complaint in the pediatric gastroenterology ward were included. Patients or their guardians who declined continued therapy and those lacking information were omitted from this study. In addition, patients with primary surgical abdominal diseases including acute appendicitis and idiopathic intussusception requiring surgical treatment were also excluded. We collected the data including age at diagnosis, gender, body mass index (BMI), intensity, duration of pain, site of pain, the common associated symptoms, final diagnosis at discharge, surgical intervention, and imaging data. For verbal children, pain site was confirmed by self-indication. For toddlers unable to describe discomfort, pain location was judged via localized abdominal tenderness, guarding and focal crying during palpation by clinicians.

All patients received blood testing and stool routine examination. The potential pathogen was explored in blood or stool sample. Samples should be cultured for bacteria and tested for viruses. Ultrasound was the primary imaging testing in the process of diagnosis. CT or endoscopic examination is often used for differential diagnosis and high suspicion of IBM or gastrointestinal tumor. However, CT was not routinely required owing to its radiation, especially for children. In addition, endoscopy was not performed in all younger children for the need of sedation. Determination of IBD or gastrointestinal tumor was based upon histological results. Diagnosis of functional gastrointestinal disorders was made according to the Rome IV Pediatric Criteria. Acute gastroenteritis presents with infectious gastrointestinal inflammation lasting < 14 days, featuring diarrhea and fever. Chronic gastritis and peptic ulcer disease are chronic conditions (pain > 3 months). Chronic gastritis was diagnosed by upper gastrointestinal endoscopy with gastric mucosal biopsy, the gold standard for confirmation. Biopsy histology showed chronic inflammatory cell infiltration in the gastric wall, and some patients tested positive for Helicobacter pylori. chronic gastritis and peptic ulcers were more common in older children with persistent epigastric pain. Endoscopic identification of mucosal craters distinguished peptic ulcers from gastritis. Chronic abdominal pain is pain that is present for > 3 months. All patients were divided into younger children (< 6 years) and older children as well as adolescents (≥ 6 years) with 6 years as the cut-off age. This age stratification is commonly applied in pediatric research. Significant differences in disease spectrum, clinical symptoms and subjective symptom description exist between preschool children and school-age children/adolescents, so this grouping method was adopted for statistical analysis. The site of pain mainly included epigastric, periumbilical, lower abdominal, diffuse pain. Concomitant symptoms in this study mainly consisted of fever, nausea, vomiting and hematochezia.

### Statistical analysis

All statistical analyses were conducted using SPSS version 14.0 (SPSS Inc., Chicago, IL, USA). The categorical variables were presented as numbers and percentages. Baseline characteristics of participants were compared via χ2 tests. Fisher’s exact test was carried out when the data distribution did not meet the applicability criteria of the Chi-square test. All continuous variables were first assessed for normality of distribution using the Shapiro-Wilk test. Continuous variables that conformed to a normal distribution were presented as mean ± standard deviation, and inter-group comparisons were carried out via the Student’s t-test. The non-normal distribution continuous variable data were expressed as median (25th percentile, 75th percentile), and the inter-group comparisons were carried out using the Wilcoxon rank-sum test. A *p-value* of < 0.05 was considered statistically significant.

## Results

This study was carried out in 1025 patients who suffered from abdominal pain. All patients underwent complete blood count and stool examinations. Bacterial acute gastroenteritis showed leukocytosis and neutrophil predominance, while viral cases presented lymphocytosis. HSP patients frequently had mild leukocytosis, thrombocytosis and elevated inflammatory markers. Most children with chronic gastritis had normal blood profiles; mild anemia occasionally occurred in acute gastric mucosal lesions due to mucosal bleeding. No hematological malignancy signs were found; patients with suspicious blood abnormalities were transferred to hematology and excluded from this cohort. For stool tests, rotavirus and norovirus were the leading viral pathogens in acute gastroenteritis, with *Campylobacter jejuni* and *nontyphoidal Salmonella* as major bacterial strains. Positive fecal occult blood was common in HSP with intestinal hemorrhage, whereas stool findings were mostly normal in chronic gastritis. In addition, abdominal ultrasound was performed routinely. Different disease groups displayed distinct imaging features: acute gastroenteritis showed diffuse intestinal edema, HSP presented bowel wall thickening plus mesenteric lymphadenopathy with three intussusception cases, and appendicitis manifested thickened appendix with peripheral effusion. Ultrasound yielded no specific abnormalities for chronic gastritis, peptic ulcers and acute gastric mucosal lesions, whose diagnosis relied on endoscopy instead. Functional gastrointestinal disorders had completely normal ultrasound results.

Among the included patients, there were 625 boys (60.98%) and 400 girls (39.02%), and the median age of this cohort was 8.0 years (5.00, 11.00). Most included patients (76.39%) had a history of acute pain. The median duration of pain was 2 months (0.1, 10.00). Only 31 (3.02%) patients presented with dehydration symptoms. This pain in most patients localized in the periumbilical region (54.63%). In addition, The most common associated symptoms were nausea and/or vomit (53.46%), diarrhea (22.63%), and fever (22.44%). 668 (65.17%) patients underwent gastrointestinal endoscopy. The detailed distribution of clinical characteristic was shown in Table 1.

**Table 1:** The clinical characteristics of children with abnormal pain

Variable		Median (range)/ *n *(%)
Age (year)	Median (Q1, Q3)	8.00 (5.00, 11.00)
Gender	Boy/Girl	625 (60.98)/400 (39.02)
BMI ( kg/m2)	Median (Q1, Q3)	15.64 (14.30, 17.99)
Intensity	Acute/Chronic	783 (76.39)/242 (23.61)
Duration of pain (month)	Median (Q1, Q3)	2.00 (0.1, 10.00)
Dehydration	Yes/No	31 (3.02)
Site of pain	Epigastric pain	402 (39.22)
	Periumbilical pain	560 (54.63)
	Lower abdominal pain	56 (5.46)
	Diffuse abdomen	7 (0.68)
The most common associated symptoms	Fever	230 (22.44)
	Diarrhea	232 (22.63)
	Nausea and/or vomit	548 (53.46)
	Hematochezia	46 (4.49)
Gastrointestinal endoscope	Yes/No	668 (65.17)

After a detailed examination, 458 cases were diagnosed with chronic gastritis, 321 with acute gastroenteritis, 100 with Henoch-Schonlein purpura (HSP), 44 with chronic enteritis, 41 with peptic ulcer disease, 21 with functional gastrointestinal disorder, 16 with appendicitis, and nine with acute gastric mucosal lesion. In addition, among 100 HSP patients, routine admission abdominal ultrasound identified 3 children complicated with intussusception. One case underwent conservative therapy, while two cases received emergency surgical manual reduction due to suspected intestinal ischemia (Table 2).

**Table 2:** The most common diagnosis of patients presenting with abdominal pain at our ward

Causes	*n* (%)
Chronic gastritis	458 (44.68)
Acute gastroenteritis	321 (31.32)
Henoch-Schonlein purpura	100 (9.76)
Peptic ulcer disease	41 (4.00)
Chronic enteritis	44 (4.29)
Functional gastrointestinal disorder	21 (2.05)
Appendicitis	16 (1.56)
Inflammatory bowel disease	11 (1.07)
Acute gastric mucosal lesion	9 (0.88)
Others	4 (0.39)

We analysed the clinical features of included patients according to disease types. The most common diseases were chronic gastritis, acute gastroenteritis and HSP. The median age was 10.00, 5.00 and 7.00 years, respectively. Most patients were male (59.39% vs. 60.75% vs. 62.00%, respectively). After a detailed analysis, statistical differences (all *P* < 0.001) were found in age, BMI, abdominal pain duration, main pain site (epigastric pain, periumbilical pain), and rates of concomitant symptoms (fever, diarrhea, nausea/vomiting) across the three groups. The detailed distributions were shown in Table 3.

**Table 3:** Clinical characteristics of patients according to disease types

Variable		Chronic gastritis	Acute Gastroenteritis	HSP	*P*-value
No of patients		458	321	100	
Age (year)	Median (Q1, Q3)	10.00 (8.00, 12.00)	5.00 (3.00, 7.00)	7.00 (5.00, 10.00	< 0.001
Gender	Boy/Girl	272 (59.39)/186 (40.61)	195 (60.75)/126 (39.25)	62 (62.00)/38 (38.00)	0.751
BMI (kg/m2 )	Median (Q1, Q3)	15.98 (14.46, 19.39)	15.15 (14.05, 16.67)	15.04 (13.46, 16.55	< 0.001
Duration of pain (month)	Median (Q1, Q3)	1.00 (0.50, 6.00)	0.06 (0.03, 0.30)	0.17 (0.10, 0.30)	< 0.001
Site of pain	Epigastric	208 (45.41)	90 (28.04)	44 (44.00)	< 0.001
	Periumbilical	225 (49.13)	213 (66.36)	52 (52.00)	< 0.001
	Lower abdominal	23 (5.02)	14 (4.36)	3 (3.00)	0.676
Concomitant symptoms	Fever	35 (7.64)	162 (50.47)	9 (9.00)	< 0.001
	Diarrhea	56 (12.23)	132 (41.12)	7 (7.00)	< 0.001
	Nausea and/or vomit	178 (38.86)	253 (78.82)	66 (66.00)	< 0.001
	Hematochezia	12 (2.62)	11 (3.43)	7 (7.00)	0.083

We analysed the clinical features of included patients according to gender. As shown in Table 4, there were 625 boys and 400 girls. The common disease types were chronic gastritis, acute gastroenteritis, HSP, and chronic enteritis in both genders. There was no significant difference in age, duration, intensity, site of pain, and concomitant symptoms between the two groups (*P* > 0.05), while boys had a significantly higher BMI than girls (*P* = 0.007).

**Table 4:** Clinical characteristics of patients according to gender

Variable		Boys	Girls	*P*-value
No of patients		625	400	
Age (year)	Median (Q1, Q3)	8.00 (5.00, 11.00)	8.00 (5.00, 10.00)	0.755
BMI (kg/m2 )	Median (Q1, Q3)	15.75 (14.48, 18.30)	15.41 (14.05, 17.63)	0.007
Duration of pain (month)	Median (Q1, Q3)	0.30 (0.10, 2.00)	0.46 (0.10, 3.00)	0.195
Intensity	Acute/Chronic	488 (78.08)/137 (21.92)	295 (73.75)/105 (26.25)	0.085
Site of pain	Epigastric	241 (38.56)	161 (40.25)	0.524
	Periumbilical	345 (55.20)	215 (53.75)	0.627
	Lower abdominal	35 (5.60)	21 (5.25)	0.807
Concomitant symptoms	Fever	133 (21.28)	97 (24.25)	0.245
	Diarrhea	148 (23.68)	84 (21.00)	0.338
	Nausea and/or vomit	343 (54.88)	205 (51.25)	0.329
	Hematochezia	33 (5.28)	13 (3.25)	0.119
Common causes		Chronic gastritis (272), acute gastroenteritis (195), HSP (62), chronic enteritis (31), peptic ulcer disease (30)	Chronic gastritis (186), acute gastroenteritis (126), HSP (38), chronic enteritis (12), peptic ulcer disease (11)	

We analysed the clinical features of included patients according to age. A total of 1025 patients were divided into young children (*n* = 272) and older children and adolescents (*n* = 753). Young children are frequently diagnosed with acute gastroenteritis, whereas chronic gastritis is the predominant diagnosis in older children and adolescents. There were significant differences in age, BMI, pain duration and pain intensity between the two groups (all *P* < 0.001), while gender distribution showed no statistical difference (*P* = 0.701). In terms of pain site, the two groups had significant differences in epigastric and periumbilical pain (*P* = 0.003, *P* = 0.001). For concomitant symptoms, the incidences of fever, diarrhea, nausea and/or vomit in young children group were significantly higher (all *P* < 0.001) (Table 5).

**Table 5:** Clinical characteristics of patients according to age

Variable		Young children	Older children and Adolescents	*P*-value
No of patients		272	753	
Age (year)	Median (Q1, Q3)	4.00 (3.00, 5.00)	9.00 (8.00, 12.00)	< 0.001
Gender	Boy/Girl	168 (61.76)/104 (38.24)	457 (60.69)/296 (39.31)	0.701
BMI (kg/m2 )	Median (Q1, Q3)	15.00 (13.88, 16.06)	15.98 (14.48, 19.10)	< 0.001
Duration of pain (month)	Median (Q1, Q3)	0.10 (0.03, 0.17)	0.75 (0.13, 4.00)	< 0.001
Intensity	Acute/Chronic	255 (93.75)/17 (6.25)	528 (70.12)/225 (29.88)	< 0.001
Site of pain	Epigastric	86 (31.62)	316 (41.97)	0.003
	Periumbilical	173 (63.60)	387 (51.39)	0.001
	Lower abdominal	12 (4.41)	44 (5.84)	0.355
Concomitant symptoms	Fever	122 (44.85)	108 (14.34)	< 0.001
	Diarrhea	89 (32.72)	143 (18.99)	< 0.001
	Nausea and/or vomit	188 (69.12)	360 (47.81)	< 0.001
	Hematochezia	12 (4.41)	34 (4.52)	0.951
Common causes		Acute gastroenteritis (180), chronic gastritis (28), HSP (28), chronic enteritis (8)	Chronic gastritis (430), acute gastroenteritis (141), HSP (72), peptic ulcer disease (38)	

We analysed the clinical features of included patients according to intensity. They were divided into the acute group (*n* = 783) and chronic group (*n* = 242). Acute gastroenteritis (*n* = 319) was the most common diagnosis in the acute group. Chronic group mainly included chronic gastritis (*n* = 192). There were significant differences in age, BMI and pain duration between the two groups (all *P* < 0.05), while gender distribution showed no statistical difference (*P* = 0.126). In terms of pain site, the incidence of periumbilical pain was significantly higher in the acute group (*P* = 0.047). For concomitant symptoms, the incidences of fever, diarrhea and nausea/or vomiting in the acute pain group were significantly higher (all *P* < 0.05) (Table 6).

**Table 6:** Clinical characteristics of patients according to intensity

Variable		Acute pain	Chronic pain	*P*-value
No of patients		783	242	
Age (year)	Median (Q1, Q3)	7.00 (5.00, 10.00)	10.00 (8.00, 12.00)	< 0.001
Gender	Boy/Girl	488 (62.32)/295 (37.68)	137 (56.61)/105 (43.39)	0.126
BMI (kg/m2 )	Median (Q1, Q3)	15.56 (14.27, 17.80)	15.96 (14.54, 19.05)	0.01
Duration of pain (Month)	Median (Q1, Q3)	0.16 (0.06, 0.68)	12.00 (6.00, 12.00)	< 0.001
Site of pain	Epigastric	299 (38.19)	103 (42.56)	0.197
	Periumbilical	441 (56.32)	119 (49.17)	0.047
	Lower abdominal	37 (4.73)	19 (7.85)	0.060
Concomitant symptoms	Fever	219 (27.98)	11 (4.55)	< 0.001
	Diarrhea	189 (25.42)	43 (17.77)	0.045
	Nausea and/or vomit	466 (59.52)	82 (33.88)	< 0.001
	Hematochezia	37 (4.73)	9 (3.72)	0.508
Common causes		Acute gastroenteritis (319), chronic gastritis (266), HSP (97), chronic enteritis (28)	Chronic gastritis (192), peptic ulcer disease (17), chronic enteritis(16), gastrointestinal disorder (5)	

## Discussion

The problem of abdominal pain represents one of the major challenges in today’s clinical practice. With improvements in technology and advancements in imaging, doctors have become skilled at diagnosing with abdominal pain. However, children are a special population who often cannot completely express their discomforts, especially in younger children. Abdominal pain among children also accounts for a significant proportion of emergency department visits. The article describes the characteristics of abdominal pain in children, which helps narrow the work-up significantly and makes an accurate diagnosis in time.

Chronic gastritis is a life-long and progressive disease. Histologically, chronic gastritis often presents with infiltration of polymorphs and chronic inflammatory cells in the gastric mucosa, along with the development of glandular atrophy and intestinal metaplasia. It is not easily cured and is prone to recurrence. Data on its incidence in children are limited, but available evidence supports a range of 2% to 8% [5, 6], with incidence increasing beyond 10 years of age [7]. Patients with chronic gastritis typically present with symptoms such as epigastric discomfort, bloating, acid regurgitation, pain, decreased appetite, and belching. The most common cause in children is Helicobacter pylori infection. Other potential etiologies of gastritis should be taken into account, including autoimmune or atrophic gastritis, eosinophilic gastritis, collagenous gastritis, and upper gastrointestinal involvement in IBD. Most Helicobacter pylori-infected individuals, including children, are asymptomatic. As childhood is critical for infection acquisition and prolonged infection increases cancer risk, early eradication is recommended before irreversible mucosal damage [8–10]. NASPGHAN guidelines limit pediatric testing to severe organic disease and eradication to ulcers, MALT lymphoma, or atrophic gastritis with intestinal metaplasia [11]. For symptomatic Helicobacter pylori-associated chronic active gastritis, treatment validity is unproven and the decision should be made by the physician in consultation with the patient and the patient’s family.

Acute gastroenteritis is a common disease in people of all ages, accounting for 1.5 million office visits in children younger than five years [12]. It is an inflammation of the stomach, small intestine, or large intestine, and usually lasts less than 14 days. The causes of acute gastroenteritis are diverse. To my knowledge, enteric viruses, particularly rotaviruses and noroviruses, are a leading cause of gastroenteritis worldwide [13]. Rotavirus strains vary by season and geographically within countries. Rotavirus infection is seasonal and reaches a peak in late winter [13]. Viruses can damage small bowel enterocytes and subsequently cause acute vomiting and watery diarrhea, often accompanied by low-grade fever and abdominal cramps. Viral gastroenteritis is generally self-limited with recovery within 2–5 days, and treatment mainly maintain adequate hydration. Inappropriately stored or processed meats and seafood are common sources of bacterial pathogens. Ingestion of contaminated food and drink containing bacterial toxins causes rapid onset of vomiting or diarrhea. Bacterial enteric pathogens tend to predominate during warmer weather. Bacterial infection mainly includes Campylobacter jejuni and Nontyphoidal Salmonella spp and can trigger inflammation of the lining of the small and large intestine [14]. Thus, children have high fever and may have blood and white blood cells in their stools, even central nervous system involvement. Bacterial pathogens occasionally spread systemically, especially in young children. It is important to assess the extent of dehydration in gastroenteritis as dehydration status determines the immediate management. The infant or child with profuse watery diarrhea and frequent vomiting is most at risk. Antibiotic administration can be recommended in patients with moderate to severe illness, particularly for patients with extra-intestinal infection. The laboratory finding useful is that samples should be cultured for bacteria and tested for viral pathogens [15]. Testing for rotavirus, norovirus, and sometimes other viruses is available in most hospitals using methods for rapid antigen detection.

Chronic enteritis is an important and underrecognized cause of abdominal pain in children. Its clinical manifestations are mostly non-specific, and the condition is closely associated with immature intestinal barrier function, intestinal dysbiosis, and immune dysregulation. Infectious, allergic, and dietary factors are common etiologies, whereas severe inflammatory bowel disease is rare in the pediatric population [16]. Due to the lack of typical symptoms, chronic enteritis is often misdiagnosed as functional gastrointestinal disorders. Long-standing intestinal inflammation may lead to visceral hypersensitivity, intestinal dysmotility, and persistent abdominal pain. For children with recurrent or chronic abdominal pain, especially those with diarrhea or growth impairment, chronic enteritis should be considered early. Comprehensive evaluation and timely intervention can effectively alleviate intestinal inflammation, relieve abdominal symptoms, and improve clinical outcomes in affected children.

HSP is a systemic vasculitis with multiorgan involvement, and accounts for 10–30/100,000 children per year under 17 years old [17, 18]. The classic presentations include purpura, arthritis or arthralgia, abdominal pain and renal disease. It has been reported that approximately two thirds of children with HSP suffer from diffuse abdominal pain, often increasing after meals and accompanied by nausea and vomiting. Abdominal symptoms and skin changes occur concurrently in patients with HSP [19]. These abdominal symptoms are usually caused by submucosal haemorrhage and oedema of the bowel wall, especially in the proximal small bowel. The most severe gastrointestinal complication is intussusception characterised by severe colicky abdominal pain in nature and vomiting. Other less common complications including gangrene of the bowel, bowel perforation and massive haemorrhage also lead to abdominal pain. Laboratory tests are complementary in assessing renal involvement (urinalysis, urine microscopy, serum creatinine), and abdominal ultrasound is used in the evaluation of its possible abdominal complications [20]. In addition, biopsy of the affected skin or kidney in patients with uncertain diagnosis is required.

In addition, other diseases also can cause abdominal pain. Neonates, infants and toddlers often present with inconsolable crying and irritability. Older children express possible site of pain. Gastritis and pancreatitis are associated with epigastric pain. Pain in the right hypochondrium is representative of hepatitis and cholecystitis. Drug intake may cause drug-induced gastritis, especially non-steroidal anti-inflammatory drugs (NSAIDs). Lumbar pain radiating to the groin may suggest nephrolithiasis, while the shifting pain in right lower quadrant may be acute appendicitis [21]. Children with functional constipation have failure to pass stools at times [22]. Traumatic abdominal pain should be considered in patients with a history of blunt trauma [23].

This study has limitations. menstrual status and premenstrual tension symptoms were not collected in the original clinical data. Therefore, we could not evaluate the correlation between premenstrual discomfort and abdominal pain in adolescent girls in this retrospective cohort. It does not propose novel pathogenic theories or innovative therapeutic strategies for childhood abdominal pain, which is an inherent limitation of single-center retrospective observational research. Nevertheless, the present study provides recent three-year clinical data of hospitalized children with abdominal pain, enriching regional epidemiological data of pediatric abdominal pain in eastern China. These findings carry practical guiding value for daily clinical diagnosis and treatment. Future prospective multi-center research will be conducted to further explore potential innovative research directions.

In conclusion, our study showed that in patients with abdominal pain, chronic gastritis was the most common disease, followed by acute gastroenteritis, HSP and chronic enteritis. Most patients presented with acute and periumbilical pain, with vomiting being the most frequent concomitant symptom. Stratified subgroup analyses by pain intensity, age, gender and disease type revealed obvious inter-group differences in pain duration, pain location and accompanying symptoms. Routine blood, stool tests and abdominal ultrasound serve as effective initial screening tools, while gastrointestinal endoscopy is essential for confirming gastric mucosal lesions. Abdominal ultrasound successfully detected intussusception complications in HSP patients. Although this is a retrospective study, it could help doctors make an accurate diagnosis in time.

## Data Availability

All data generated or analysed during this study are included in this published article.

## Abbreviations

HSP: Henoch-Schönlein purpura
IBS: Irritable bowel syndrome
CP: Chronic pancreatitis
IBD: Inflammatory bowel disease
BMI: Body mass index
NSAIDs: Non-steroidal anti-inflammatory drugs
